# Elevated lactate/albumin ratio is associated with poor prognosis in sepsis patients: A systematic review and meta-analysis

**DOI:** 10.5937/jomb0-42284

**Published:** 2024-06-15

**Authors:** Xian Zhao, Qin Peng, Weiwei Li, Dongmei Hu, Yue Guan, Jingwen Wang

**Affiliations:** 1 Fourth Military Medical University, Xijing Hospital, Department of Pharmacy, Xi'an, China; 2 Fourth Military Medical University, Xijing Hospital, Department of Hepatobiliary Surgery, Xi'an, China

**Keywords:** lactate/albumin ratio, prognosis, sepsis, meta-analysis, odnos mlečne kiseline/albumina, prognoza, sepsa, meta-analiza

## Abstract

**Background:**

The aim of this study was to explore the association between lactate/albumin ratio and the prognosis of sepsis patients.

**Methods:**

A computerized search was performed in Pubmed, EMbase, Ovid, Medline, and Google Scholar to collate relevant studies. The results were compared using standardized mean differences (SMD)/odds ratio (OR) and 95% confidence intervals (CI). Prospective and retrospective cohort studies were both included in this study.

## Abstract (sr)

**Uvod:**

Cilj ove studije je istraživanje veze između odnosa mlečne kiseline/albumina i prognoze kod pacijenata sa sepsom.

**Metode:**

Izvršena je kompjuterska pretraga u bazama podataka Pubmed, EMbase, Ovid, Medline i Google Scholar kako bi se sakupile relevantne studije. Rezultati su upoređeni koristeći standardizovane srednje razlike (SMD)/odnose verovatnoće (OR) i 95% intervala poverenja (CI). Ova studija uključuje i prospektivne i retrospektivne kohortne studije.

**Rezultati:**

Uključeno je ukupno devet studija sa 3039 učesnika. Analiza pokazuje da su preživeli imali znatno niži odnos mlečne kiseline/albumina u poređenju sa onima koji nisu preživeli (SMD=-2,02, 95% CI: -2,76 do -1,28, I 2=97,4%). Takođe, naši rezultati ukazuju da povišen odnos mlečne kiseline/albumina predstavlja nezavisni faktor rizika za smrtnost (OR=2,16, 95% CI: 1,58 do 2,95, I 2=76,2%) i sindrom disfunkcije više organa (MODS) (OR=3,41, 95% CI: 1,78 do 6,50, I2=0,0%) kod pacijenata sa sepsom. Takođe, prema rezultatima površine ispod krive (AUC), odnos mlečne kiseline/albumina takođe pokazuje dobru diskriminativnu moć u predviđanju smrtnosti (AUC=0,75, 95% CI: 0,68 do 0,84, I2=92,9%) i MODS (AUC=0,78, 95% CI: 0,68 do 0,91, I2=65,1%) kod pacijenata sa sepsom. Begov i Egerov test ukazuju na odsustvo pristrasnosti kod uključenih studija.

**Zaključak:**

Naši rezultati ističu da je odnos mlečne kiseline/albumina važan prognostički faktor za sindrom disfunkcije više organa (MODS) i smrtnost kod pacijenata sa sepsom, sa dobrim sposobnostima za identifikaciju MODSa i smrtnosti. Povišen odnos mlečne kiseline/albumina predstavlja nezavisni faktor rizika za smrtnost i sindrom disfunkcije više organa (MODS) kod pacijenata sa sepsom. Takođe, odnos mlečne kiseline/albumina pokazuje dobru diskriminativnu moć za predviđanje smrtnosti i MODS-a kod pacijenata sa sepsom.

## Introduction

Sepsis and septic shock are notable causes of organ dysfunction because of tissue hypoperfusion and hypoxia, in turn leading to life-threatening emergencies [1] [2] [3] [4]. Presently, sepsis remains the principal cause of intensive care unit (ICU) admission and is regarded as a major disease of concern by global healthcare professionals [5] [6]. Despite significant advances in intensive care and treatment of sepsis, results from prior studies indicated that sepsis and infectious diseases are still a major concern for critical physicians [7] [8]. Additionally, previous studies revealed that regardless of said progress in intensive care and sepsis treatment strategies, the mortality rate of sepsis and septic shock is approximately 20–30%, accounting for 30–50% of in-hospital mortality [9] [10].

Sepsis patients suffer from peripheral tissue hypoxia due to inadequate oxygen supply, promoting anaerobic metabolic processes and ultimately increasing lactate concentration [11] [12]. In current clinical practice, the lactate level is frequently used to detect the degree of tissue hypoxia and, following this, guide the clinical treatment strategy and estimate the prognosis of sepsis patients [13] [14] [15]. Meanwhile, albumin is a vital serological index reflecting the severity of inflammation [16] [17]. Studies have demonstrated that albumin is a negative acute phase protein [18], which can be utilized as a useful parameter to evaluate the mortality and prognosis of various diseases [19] [20].

Recent related studies have evidenced that lactate and albumin levels are closely linked to the prognosis of sepsis patients. However, this poses the question of whether the combination of lactate and albumin, i.e., lactate/albumin ratio, can further enhance the value of predicting the prognosis of sepsis patients. Thus, this study aimed to explore the association between the lactate/albumin ratio and the prognosis of sepsis patients using meta-analysis.

## Materials and methods

This systematic review followed the PRISMA guidelines [21]. Informed consent is not required for this study as it is based on the secondary analysis of previous data. The study protocols were not registered on any website, and data supporting this study is available from public databases.

### Retrieval strategy

Related databases were searched for articles on the prognosis of lactate/albumin ratio and sepsis patients, including Pubmed, Embase, Ovid, Medline, and Google Scholar. The retrieval time was limited from the establishment of the database to May 2022. Further, intending to avoid missing crucial studies, we manually searched the references of the included studies. The key terms used in the retrieval strategy were lactate/albumin ratio, sepsis, severe sepsis, »sepsis, severe,« and septic. PubMed’s retrieval strategy was as follows:

((»Sepsis«[Mesh] OR [severe sepsis«[Mesh]) OR (»sepsis, severe »[Mesh]) OR »septic shock»[Mesh]) AND (»lactate/albumin ratio« [MeSH Terms] OR »lactate/ albumin ratio« [All Fields]). The detailed search strategy is described in Table 1.

**Table 1:** The detailed search strategy used in PubMed.

**#1**<br>»Sepsis«[Title/Abstract] OR »bloodstream infection«[Title/Abstract] OR »bloodstream infections«[Title/Abstract] OR »infection bloodstream«[Title/Abstract] OR »Pyemia«[Title/Abstract] OR »Pyemias«[Title/Abstract] OR »Pyohemia«[Title/Abstract] OR »Pyohemias«[Title/Abstract] OR »Pyaemia«[Title/Abstract] OR »Septicemia«[Title/Abstract] OR »Septicemias«[Title/Abstract] OR »blood poisoning«[Title/Abstract] OR »blood poisonings«[Title/Abstract] OR ((»poisoned«[All Fields] OR »Poisoning«[MeSH Terms] OR »Poisoning«[All Fields] OR »Poisonings«[All Fields] OR »Poisoning«[MeSH Subheading] OR »poisonous«[All Fields] OR »poisons«[Pharmacological Action] OR »poisons«[MeSH Terms] OR »poisons«[All Fields] OR »poison«[All Fields]) AND »Blood«[Title/Abstract]) OR »poisoning blood«[Title/Abstract] OR »severe sepsis«[Title/Abstract] OR »sepsis severe«[Title/Abstract]			
**#2**<br>»Lactate albumin ratio«[Title/Abstract] OR »lactate albumin«[Title/Abstract] OR »lactate to albumin ratio«[Title/Abstract] OR »lactate to albumin«[Title/Abstract] OR »lactate albumin ratio«[Title/Abstract] OR »lactate albumin«[Title/Abstract]			
**#3: #1 and #2**<br>(»Sepsis«[Title/Abstract] OR »bloodstream infection«[Title/Abstract] OR »bloodstream infections«[Title/Abstract] OR »infection bloodstream«[Title/Abstract] OR »Pyemia«[Title/Abstract] OR »Pyemias«[Title/Abstract] OR »Pyohemia«[Title/Abstract] OR »Pyohemias«[Title/Abstract] OR »Pyaemia«[Title/Abstract] OR »Septicemia«[Title/Abstract] OR »Septicemias«[Title/Abstract] OR »blood poisoning«[Title/Abstract] OR »blood poisonings«[Title/Abstract] OR ((»poisoned«[All Fields] OR »Poisoning«[MeSH Terms] OR »Poisoning«[All Fields] OR »Poisonings«[All Fields] OR »Poisoning«[MeSH Subheading] OR »poisonous«[All Fields] OR »poisons«[Pharmacological Action] OR »poisons«[MeSH Terms] OR »poisons«[All Fields] OR »poison«[All Fields]) AND »Blood«[Title/Abstract]) OR »poisoning blood«[Title/Abstract] OR »severe sepsis«[Title/Abstract] OR »sepsis severe«[Title/Abstract]) AND (»lactate albumin ratio«[Title/Abstract] OR »lactate albumin«[Title/Abstract] OR »lactate to albumin ratio«[Title/Abstract] OR »lactate to albumin«[Title/Abstract] OR »lactate albumin ratio«[Title/Abstract] OR »lactate albumin«[Title/Abstract])			

### Inclusion criteria and exclusion criteria

Inclusion criteria: patients diagnosed with sepsis without age limitation; Prospective or retrospective cohort study; the association between lactate/albumin ratio and prognosis of patients with sepsis was reported; relevant information can be extracted for subsequent analysis. Exclusion criteria: case-control studies, case reports, editorials, and letters; animal studies; studies with missing information or where data is unable to be extracted for subsequent analysis.

### Literature screening and data extraction

Two independent reviewers read the title and abstract of the literature following the retrieval strategy and keywords. They ultimately determined whether the literature was to be included in this study by reading the full text. In the case of a dispute between the two researchers, disagreements were resolved through discussion or with a third author. The study authors were contacted for clarification or additional data when necessary. The extracted basic feature information includes the first author, year of publication, country of publication, research type, recruitment time, total sample size, age, and outcome.

### Quality evaluation of included studies

The Newcastle-Ottawa Scale (NOS) was used in this study to evaluate the quality of the included studies, with a maximal score of nine. Detailed NOS scores are denoted in previous studies [22] [23].

### Statistical analysis

We utilized stata11.0 software and a random effect model to analyze the results and calculate the standardized mean differences (SMD)/odds ratio (OR) and 95% confidence intervals (CI). Regarding the measurement of lactate and albumin, the measurement methods of the included studies were not wholly consistent. For this reason, we calculated SMD with 95% CI. P value<0.05, meaning there is statistical significance. Additionally, I^2^ was calculated to determine the heterogeneity between the included studies. If I^2^>50%, this indicated significant heterogeneity between the included studies. Regarding the results with significant heterogeneity (I^2^>50%), sensitivity analysis was adopted to explore the primary sources of heterogeneity. Owing to the limited number of included studies, subgroup analysis was not conducted. In addition, Begg’s and Egger’s tests were employed to identify whether there was publication bias among the included literature.

## Results

### Flow chart of this study

**Figure 1:**
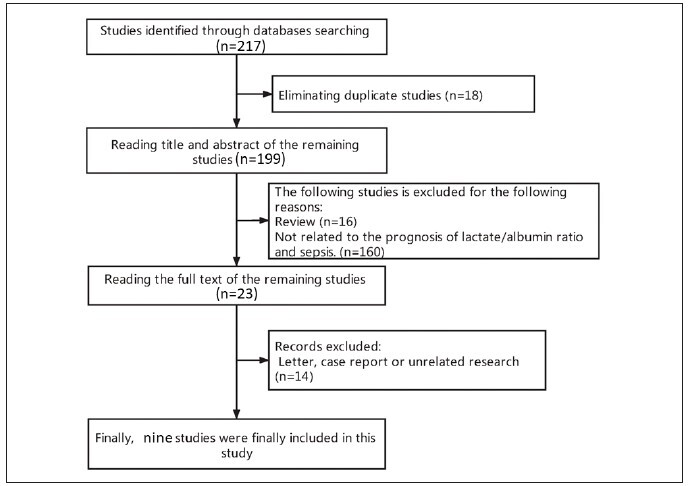
Differences in RBPs measured in EDTA+4°C during five days. Rounded data points indicate the day when bacterial contamination was observed. TG – triglycerides; TC – total cholesterol.

First, according to the initial retrieval strategy, 217 related studies were retrieved. Second, endnote software was utilized to eliminate 18 duplicate studies, leaving 199 studies. Third, after reading the titles and abstracts, 176 unrelated articles were excluded. Fourth, by reading the full text of the remaining studies, 14 studies were subsequently eliminated. Ultimately, nine studies [24] [25] [26] [27] [28] [29] [30] [31] [32] involving 3039 participants were included in this study. The flow chart is exhibited in Figure 1.

### Basic features of the included studies

The basic characteristics of the included studies are denoted in Table 2. Based on the description in Table 2, two studies are from Korea, one from Germany, one from Egypt, one from Nepal, one from Spain, one from China, one from Lebanon, and the last from Turkey. Further, the sample size of the included studies ranges from 30 to 946, while the NOS scores range from 6 to 7.

**Table 2:** Baseline characteristics of the included studies. NOS=Newcastle-Ottawa Scale

Author	Year	Country	Patients	Type of study	Recruitment time	Total<br>sample<br>sizze	Age	Outcomes	NOS<br>scores
Choi [24]	2016	Korea	Pediatric septic <br>shock patients	Retrospective <br>study	From <br>February 2012 <br>to May 2015	90	112±85.7 <br>months	28-day <br>hospital <br>mortality	7
Lichtenauer [25]	2017	Germany	Septic <br>Patients <br>Admitted <br>to ICU	Retrospective <br>study	Between <br>May 2013 and <br>November 2013	348	64.97±14.00 <br>years	In-hospital <br>mortality and <br>long-term <br>mortality	7
Moustafa [26]	2018	Egypt	Severe sepsis <br>in a pediatric <br>intensive <br>care unit	Prospective <br>cohort study	From <br>January 2016 <br>to March 2017	119	13.79±20.62 <br>months	Multiple organ <br>dysfunction syndrome and <br>mortality	7
Shin [27]	2018	Korea	Critically ill sepsis <br>patients	Retrospective <br>observational <br>study	Between <br>October 2015 and <br>February 2017	946	70.4 <br>(60.2–78.3) <br>years	28-day <br>mortality	4
Thapa [28]	2017	Nepal	Severe sepsis <br>and septic shock <br>patients	Prospective, <br>cross-sectional <br>study	From <br>November 2015 <br>to October 2016	240	Age ≥18 <br>years	Mortality	6
Trujillo [29]	2018	Spain	Sepsis and septic <br>shock patients	Historical <br>cohort study	Unclear	30	63±10 <br>years	Mortality	7
Wang [30]	2015	China	Severe sepsis <br>and septic <br>shock patients	Prospective <br>cohort study	From October 1, <br>2012, to September <br>30, 2013	54	74 <br>(68.75–80.25) <br>years	Multiple organ <br>dysfunction <br>syndrome <br>and mortality	7
Chebl [31]	2021	Lebanon	Septic <br>patients	Prospective <br>cohort study	Between <br>September 2018 <br>and February 2021	939	72.39±15.62 <br>years	In-hospital <br>mortality	7
Erdoğan [32]	2022	Turkey	Patients with <br>pneumosepsis <br>in intensive <br>care units	Retrospective <br>cohort study	Between 2018 <br>and 2020	273	71 (64–77) <br>years	In-hospital <br>mortality	7

### Comparison of lactate/albumin ratio between survivors and non-survivors

**Figure 2:**
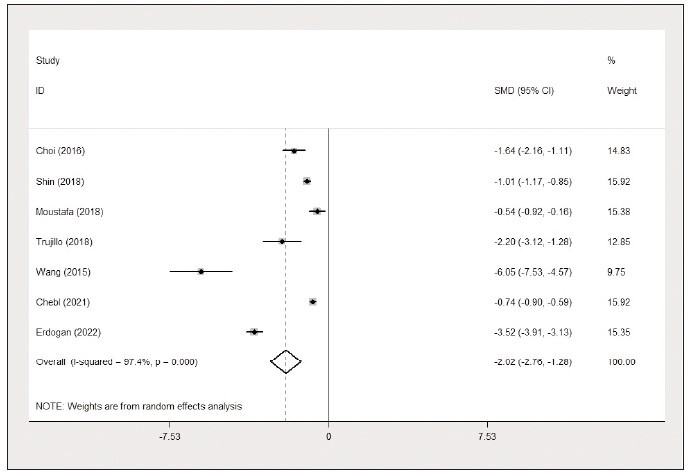
Comparison of the lactate/albumin ratio between survivors and non-survivors (forest plot).

**Figure 3:**
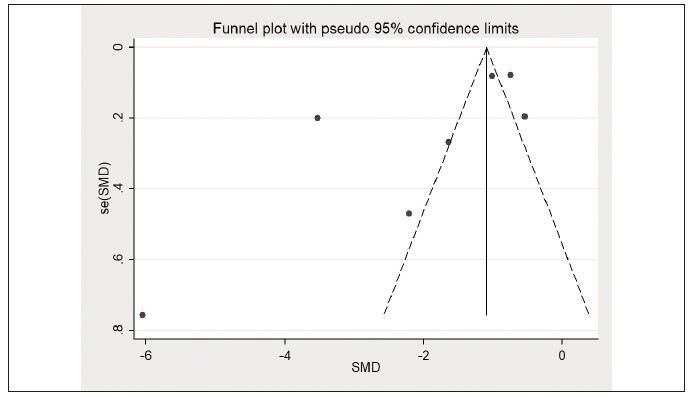
Comparison of the lactate/albumin ratio between survivors and non-survivors (funnel plot).

**Figure 4:**
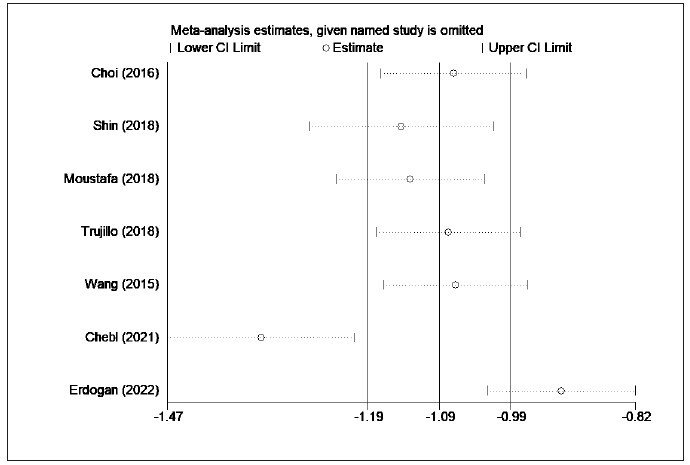
Comparison of the lactate/albumin ratio between survivors and non-survivors (Sensitivity analysis).

**Figure 5:**
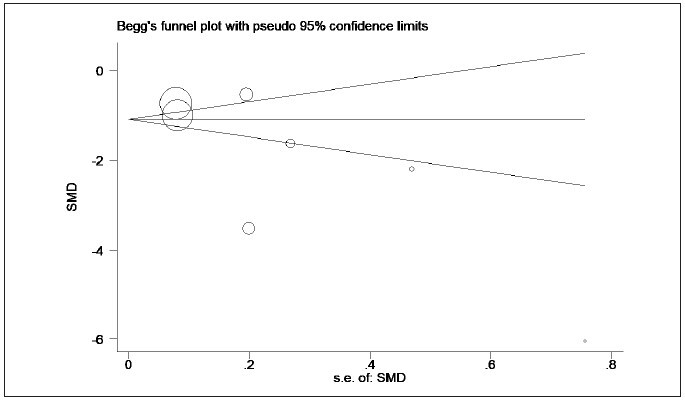
Comparison of the lactate/albumin ratio between survivors and non-survivors (Begg’s test).

Seven studies [24] [26] [27] [29] [30] [31] [32] detailed the lactate/albumin ratio levels between survivors and non-survivors. Pooled analysis revealed that survivors had substantially lower lactate/albumin ratio than non-survivors (SMD=-2.02, 95% CI: -2.76 to -1.28, I^2^=97.4%) (Figure 2), and the funnel plot is presented in Figure 3. Sensitivity analysis was further adopted to explore the source of heterogeneity on account of the obvious heterogeneity (I2=97.4%) among the included studies. Sensitivity analysis implied that the source of heterogeneity was mainly from the study of Bou Chebel et al. [31] and Erdoğan et al. [32] (Figure 4). Moreover, no publication bias was found in the included studies (Figure 5, the p-value for Begg’s and Egger’s test was 0.072 and 0.119, respectively).

### Elevated lactate/albumin ratio is associated with higher mortality

**Figure 6:**
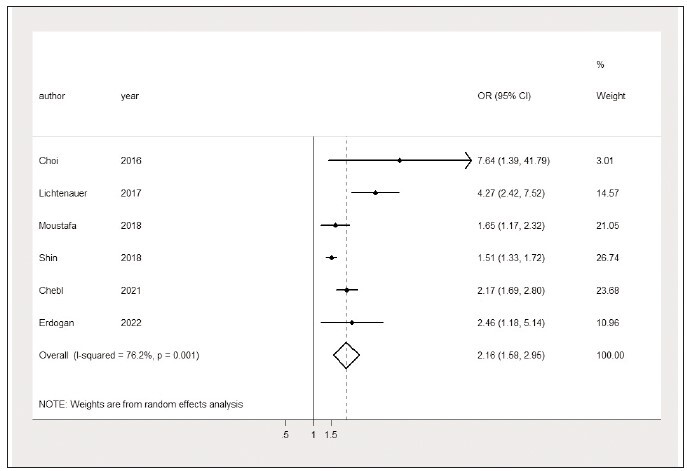
Elevated lactate/albumin ratio is associated with higher mortality (forest plot).

**Figure 7:**
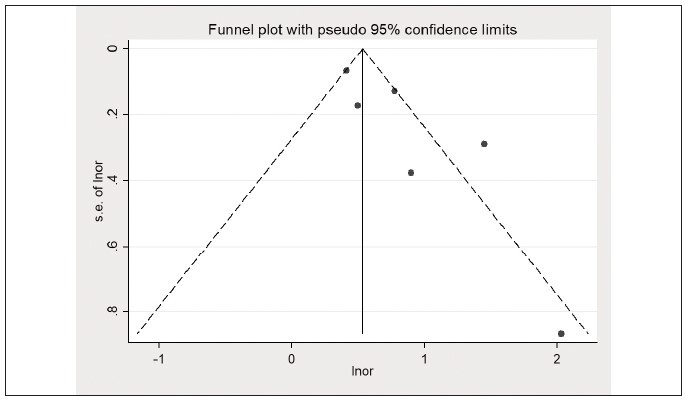
Elevated lactate/albumin ratio is associated with higher mortality (funnel plot).

**Figure 8:**
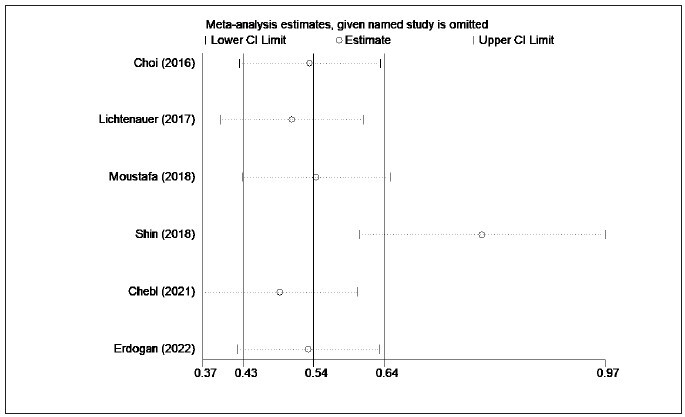
Elevated lactate/albumin ratio is associated with higher mortality (sensitivity analysis).

**Figure 9:**
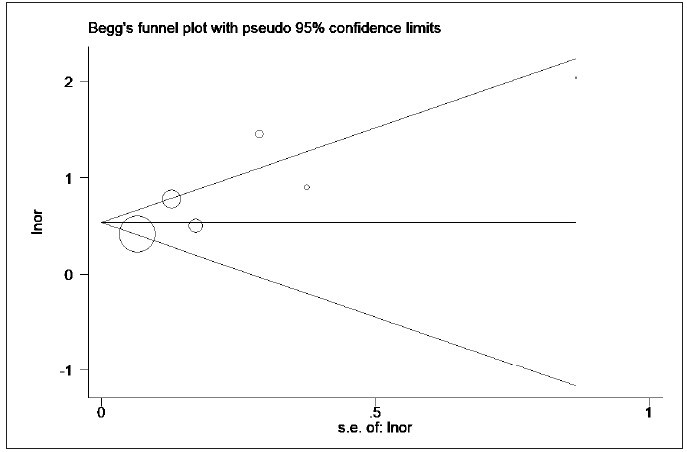
Elevated lactate/albumin ratio is associated with higher mortality (Begg’s test).

Predicated on the control of confounding factors, six studies [24] [25] [26] [27] [31] [32] evaluated whether lactate/ albumin ratio could be used as an independent risk factor for mortality in sepsis patients. The summary of the analysis results highlighted that an elevated lactate/albumin ratio is associated with higher mortality in sepsis patients (OR=2.16, 95% CI: 1.58 to 2.95, I^2^=76.2%) (Figure 6). The funnel plot is shown in Figure 7. Taking into account the heterogeneity between sxtudies, a sensitivity analysis was further performed. As shown in Figure 8, the sensitivity analysis suggested that group heterogeneity was mainly derived from the Shin et al. [27] Bou Chebel et al. [31] study. Besides, no significant publication bias was identified (Figure 9, the p-value for Begg’s and Egger’s was 0.348 and 0.064, respectively).

### Elevated lactate/albumin ratio is associated with higher risk of multiple organ dysfunction syndrome (MODS)

**Figure 10:**
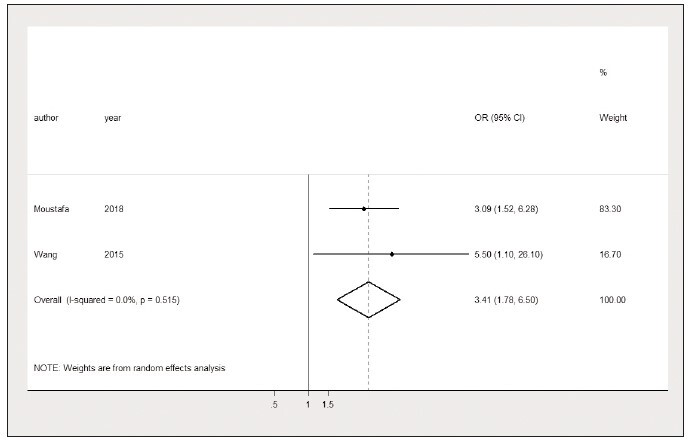
Elevated lactate/albumin ratio is associated with a higher risk of multiple organ dysfunction syndrome.

Whether the lactate/albumin ratio could be used as a prognostic factor for MODS in sepsis patients was outlined by two studies [26] [30]. Comprehensive analysis reported that after controlling for potential confounding factors, the lactate/ albumin ratio is an important prognostic factor in predicting MODS in sepsis patients (OR=2.16, 95% CI: 1.78 to 6.50, I^2^=0.0%) (Figure 10). Considering the small heterogeneity (I^2^=0.0%), sensitivity analysis and a publication bias test were not conducted.

### The area under the curve (AUC) of the lactate/albumin ratio in predicting mortality and MODS in sepsis patients

**Figure 11:**
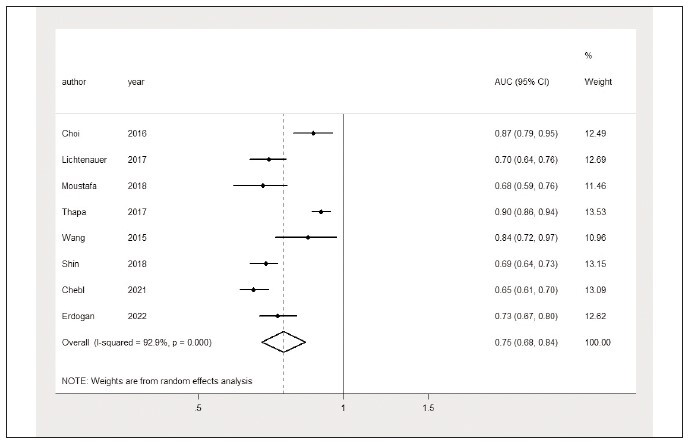
The area under the curve of the lactate/albumin ratio in predicting mortality in sepsis patients.

**Figure 12:**
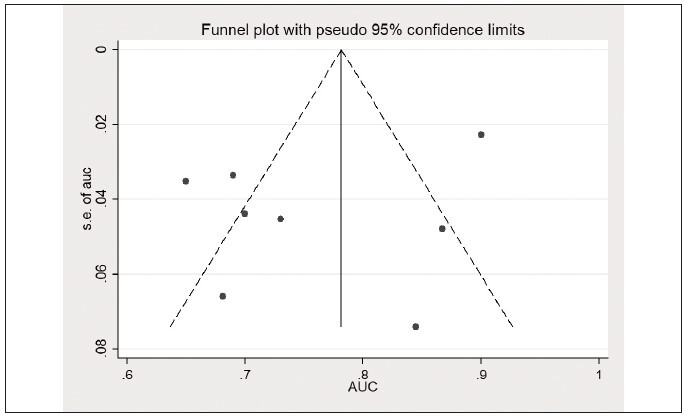
The area under the curve (AUC) of the lactate/albumin ratio in predicting mortality in sepsis patients (funnel plot).

**Figure 13:**
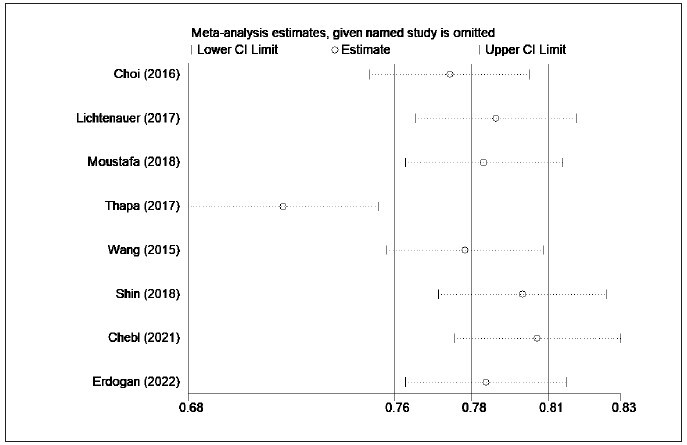
The area under the curve (AUC) of the lactate/albumin ratio in predicting mortality in sepsis patients (Sensitivity analysis).

**Figure 14:**
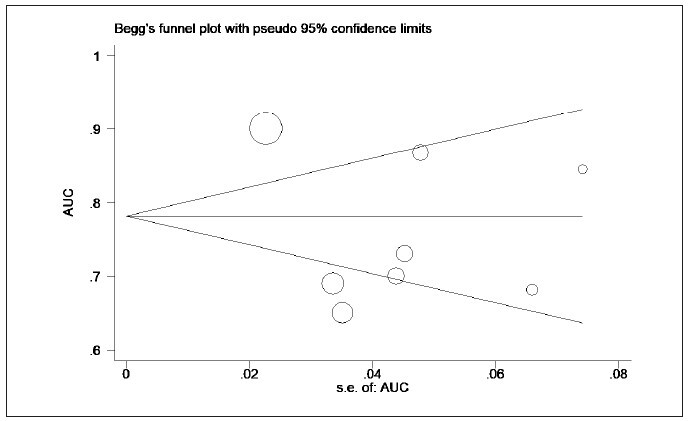
The area under the curve (AUC) of the lactate/albumin ratio in predicting mortality in sepsis patients (Begg’s test).

Eight studies [24] [25] [26] [27] [28] [30] [31] [32] disclosed the use of the AUC of the lactate/albumin ratio in predicting mortality in sepsis patients according to the receiver operating characteristic curve. The comprehensive analysis results revealed that the lactate/albumin ratio demonstrates a good discriminatory power to predict mortality in sepsis patients (AUC=0.75, 95% CI: 0.68 to 0.84, I^2^=92.9%) (Figure 11). The funnel plot is shown in Figure 12. Sensitivity analysis revealed the heterogeneity among studies, mainly from the Thapa et al. study (Figure 13). Further analysis suggested no significant publication bias among the included studies (Figure 14, the p-value for Begg’s and Egger’s was 0.62 and 0.305, respectively). In addition, the optimal lactate/ albumin ratio threshold for predicting morality is 0.775±0.473.

**Figure 15:**
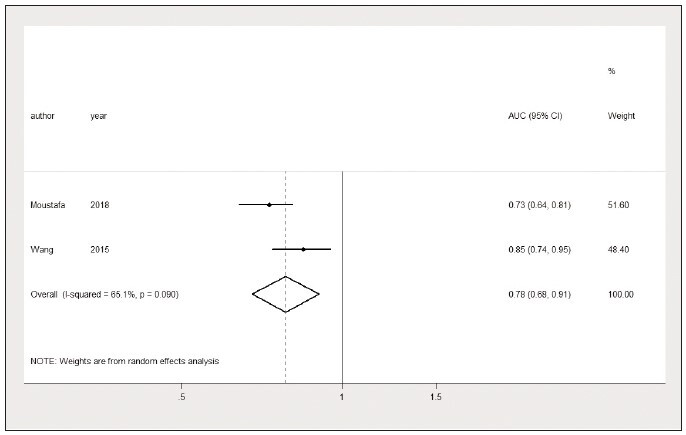
The area under the curve (AUC) of the lactate/albumin ratio in predicting multiple organ dysfunction syndrome in sepsis patients.

Likewise, we discovered that the lactate/albumin ratio also has a higher discriminatory power in predicting MODS in sepsis patients (AUC=0.78, 95% CI: 0.68 to 0.91, I^2^=65.1%) (Figure 15). Since only two studies reported the lactate/albumin ratio value in predicting MODS, no sensitivity analysis and publication bias were performed. The optimal lactate/ albumin ratio threshold for predicting MODS is 1.452±0.399.

## Discussion

As far as we know, this study was the first to evaluate the association between the lactate/albumin ratio and the prognosis of sepsis patients through meta-analysis. The findings of this study are as follows: first, our results demonstrated that for sepsis patients, the lactate/albumin ratio in survivors is substantially lower than that in non-survivors. Second, we discovered that the lactate/albumin ratio can be used to predict mortality and MODS in sepsis patients, implying that the lactate/albumin ratio can be employed in clinical practice to refine the risk stratifistratification of sepsis patients further. Third, further analysis revealed that the lactate/albumin ratio was highly differentiated in predicting mortality and MODS in sepsis patients.

Sepsis, as a kind of inflammatory disease [33], is a serious threat to life and health. Although the improvement of drug treatments and supportive treatments have improved the survival rate of sepsis patients, the mortality rate of severe sepsis remains between 25% and 30%, while the mortality rate of septic shock remains between 40% and 70% [34]. For these reasons, it is integral to identify and refine the risk stratification of sepsis early to improve the prognosis of patients. Previous studies have revealed that advanced age [35], malnutrition [36], the combination of other chronic diseases [37], and the use of immunosuppressive drugs [38] are the prognostic factors of sepsis. Yet, the mechanism of high mortality in sepsis patients cannot be fully clarified.

A retrospective cohort study of 348 sepsis patients highlighted that an elevated lactate/albumin ratio was significantly associated with adverse outcomes, even after the adjustment of confounding factors. Hence, the lactate/albumin ratio can be employed as a prognostic parameter to distinguish the risk stratification of sepsis patients [25]. Furthermore, a cohort study of 946 sepsis patients revealed that the lactate/albumin ratio was superior to lactate in predicting 28-day mortality. At the same time, this study also indicated that the lactate/albumin ratio could be a useful prognostic factor for sepsis patients regardless of the initial lactate, liver, and kidney function levels [27]. Other studies have similarly indicated that the lactate/albumin ratio could be used to predict poor prognosis in pediatric septic shock patients [24] [26], implying that the lactate/albumin ratio could also be used as a predictor of poor prognosis in pediatric or adult sepsis patients. The results of our meta-analysis are consistent with those of previous results. Moreover, founded on the results of the AUC, our research also revealed that the lactate/albumin ratio demonstrates a good discriminatory power to predict mortality (AUC=0.75, 95% CI: 0.68 to 0.84) and MODS (AUC=0.78, 95% CI: 0.68 to 0.91). The results of this study imply that clinicians should shift attention towards the lactate/albumin ratio to further refine the risk stratification of sepsis and guide treatment strategies.

Traditionally, it was thought that elevated lactate levels in septic patients were caused by a lack of oxygen delivery, which increased anaerobic glycolysis levels [39] [40]. The theory behind this concept is that anaerobic glycolysis is the primary source of lactic acid increase. However, after extensive medical studies, scientists determined that a series of factors cause hyperlactatemia. A review indicated that shock, local tissue ischemia, diabetic ketoacidosis, and anaerobic muscle activity are closely related to an elevated lactate level [41]. As well as a lack of oxygen and nutrition extraction in peripheral tissues, septic shock is commonly linked to the dysfunction of large circulation and microcirculation [42]. Lactate has become a useful marker of tissue hypoperfusion, and clinicians often guide fluid resuscitation and use inotrope/vasopressor drugs in sepsis patients following the lactate level. Additionally, the mechanism of sepsis-related hyperlactatemia is not specific, possibly because of the presence of oxygen debt or low perfusion in the tissues, resulting in an increase of anaerobic digestion and, eventually, plasma lactate production.

Further, this may be the result of insufficient lactate clearance in plasma and increased aerobic glycolysis in skeletal muscle by adrenaline [43]. On the other hand, albumin is a reliable marker of body fragility, high sensitivity to stressors, and unstable internal environment. It is also related to the prognosis of critically ill patients [44].

The following limitations exist in our meta-analysis. First, owing to the limitations of the literature, subgroup analysis was not conducted pursuant to the country, nationality, and population to further explore the source of heterogeneity. Second, the treatment strategy is closely linked to the prognosis of sepsis patients. Yet, some of the included literature did not describe the treatment strategy in detail, potentially leading to increased mixed bias and high heterogeneity. Third, several of the included studies did not stipulate the etiology of sepsis, which might also lead to higher mixed bias. Fourth, it would be interesting to compare the value of lactate/albumin ratio and lactate in predicting the prognosis of patients with sepsis. However, due to the limited data and published literature presently, no comparative study could be carried out. Fifth, the sample size of Shin’s study [27] is substantially larger than that of other studies, potentially being an important reason for the high heterogeneity. Finally, there were significant differences in age between these two pediatric studies (112±85.7 months versus 13.79±20.62 months, as can be seen in Table 2), limiting further subgroup analyses. Therefore, the conclusions of this study should be used with caution in pediatric research.

## Conclusion

Our results indicated that the lactate/albumin ratio is a vital prognostic factor for MODS and mortality in sepsis patients and has a good ability to identify MODS and mortality. Clinicians must pay close attention to the lactate/albumin ratio to refine the risk stratification of sepsis patients and adjust the treatment strategy in time to improve the prognosis of patients.

## Dodatak

### Acknowledgments

None.

### Data Availability

The data used to support the findings of this study are included in the article.

### Funding

None.

### Conflict of interest statement

All the authors declare that they have no conflict of interest in this work.
